# Effect of Polydimethylsiloxane Oil Lubrication on the Friction of Cross-Country UHMWPE Ski Bases on Snow

**DOI:** 10.3389/fspor.2022.894250

**Published:** 2022-07-05

**Authors:** Audun Formo Buene, Sondre Bergtun Auganæs, Alex Klein-Paste

**Affiliations:** Department of Civil and Environmental Engineering, Norwegian University of Science and Technology, Trondheim, Norway

**Keywords:** silicone oils, ski-snow friction, linear tribometer, skiing, lubrication, fluorine free, ski wax

## Abstract

Silicone oils are known for their excellent lubricating properties, low toxicity and are ice-, snow-, and hydrophobic. With the upcoming ban on fluorine-containing glide products imposed by the International Ski Federation (FIS), novel glide enhancers for skis are desperately needed. Here, the effect of four silicone oil viscosities (10, 20, 50, and 100 cSt) have been evaluated at three temperatures and snow conditions ranging from −10 °C dry snow to +5 °C wet snow. In dry snow conditions, the shear forces introduced by the silicone oil film increased friction significantly compared to a ski without any treatment. On wet snow, the increased hydrophobicity from the silicone oils reduced the friction by 10%. While commercial glide wax outperformed the silicone oils, this study reports the silicone oils do have desirable friction reducing properties for wet conditions.

## Introduction

Fluorinated ski glide waxes have been the state of the art in reducing ski-snow friction for decades. Their unrivaled hydrophobic properties made such products a necessity for ambitious racers. However, the waxers preparing the skis with fluorinated compounds are risking severe health effects such as decreased fertility, cancer, and hormone imbalance (Freberg et al., 2010; Vieira et al., 2013). Per- and polyfluoroalkyl substances (PFAS) have also been shown to bio-accumulate in nature (Plassmann and Berger, 2013; Grønnestad et al., 2019). Therefore, fluorinated glide products are currently being banned from ski waxes by the International Ski Federation (FIS), and their production is increasingly tightly regulated by the European Union and other authorities. Needless to say, the skiing community is in dire need of fluorine-free glide products with competitive performance (Almqvist et al., 2022).

Silicones are synthetic compounds where a silicone and oxygen backbone carry sidechains made from carbon and hydrogen. The simplest silicone polymer is polydimethylsiloxane (PDMS), and the viscosity can be controlled by altering the polymer chain lengths (Barca et al., 2014). By replacing one or both of the methyl groups, the chemical and physical properties of the polymer may be changed. Colloquially, short-chained polymers are referred to as silicone oils and longer ones as grease. If the polymers are chemically crosslinked, silicone rubbers or caulks are made.

The hydrophobic nature of PDMS silicone oils stems from the organic substituents on the silicone backbone and the free rotation of the methyl groups. The silicone-oxygen bond is extremely strong, making silicone products exceptionally resilient to both high and low temperatures. PDMS is considered non-toxic and is used as anti-foaming additives in foods, cosmetics, and other domestic applications. Silicone oils have previously been used in reduced ice adhesion applications (Yeong et al., 2016), as lubricants for hard icephobic coatings (Zheng et al., 2022) and in self-repairing slippery liquid-infused porous surfaces (SLIPS) (Yuan et al., 2022).

The friction between sliding objects and snow is inherently complex and highly dependent on snow parameters such as temperature, grain size and humidity (Theile et al., 2009). Under dry conditions, the strategy to reduce friction, according to the prevailing self-lubrication theory of ski-snow friction, is to minimize contact area and create a lubricating water film by friction melting. On wet snow, increased hydrophobicity and the ability to disrupt and minimize the water film will reduce suction from capillary bridges, thus reducing friction (Colbeck, 1992). While the meltwater-lubricated friction theory is well-established, an alternative friction model for sliders on snow where dry-contact abrasion is the major frictional contributor at low speeds (0.36–1.40 ms^−1^) and pressures (0.8–4.5 kPa) has been thoroughly investigated by Lever et al. (2019).

For skiing, friction-reducing efforts have focussed on structuring the UHMWPE base material by grinding (Rohm et al., 2015, 2016), imprinting (Breitschädel et al., 2010; Nordin and Styring, 2014), and sanding (Giesbrecht et al., 2010). The development of glide waxes has been industry-driven, and little has reached the scientific literature. Analytical efforts have been made to assess, understand, and classify commercial glide waxes (Rogowski et al., 2005; Breitschädel et al., 2014; Rohm et al., 2017) and prior to the health concerns of fluorinated compounds, the effect of fluorine content on the water-repellence of glide wax was investigated (Rogowski et al., 2007).

Curable silicone coatings for skiing applications have previously been patented and commercialized (Prince et al., 2015). To the best of the authors' knowledge, such products have not yet reached a performance level necessary for racing and are mostly enjoyed in recreational skiing. Silicone sprays were also common in the early days of classic “rub-skis” to keep water from freezing in the sandpaper-rubbed grip zone. Several commercial glide products contain silicone compounds, but we are not aware of any studies of pure PDMS silicone oils effect on ski-snow friction.

In this study, we investigate the effect of pure polydimethylsiloxane oil lubrication of UHMWPE-snow interactions at different snow conditions and temperatures. As silicone oils are reported to completely spread on a polyethylene surface (Fox and Zisman, 1952), we hypothesized the hydrophobicity and free rotation of the PDMS methyl groups will reduce the friction of skis under wet conditions. Under dry conditions, we were curious about the lubricating ability of silicone oils on the friction between contact points.

## Materials and Methods

### Materials

**Figure 1:**
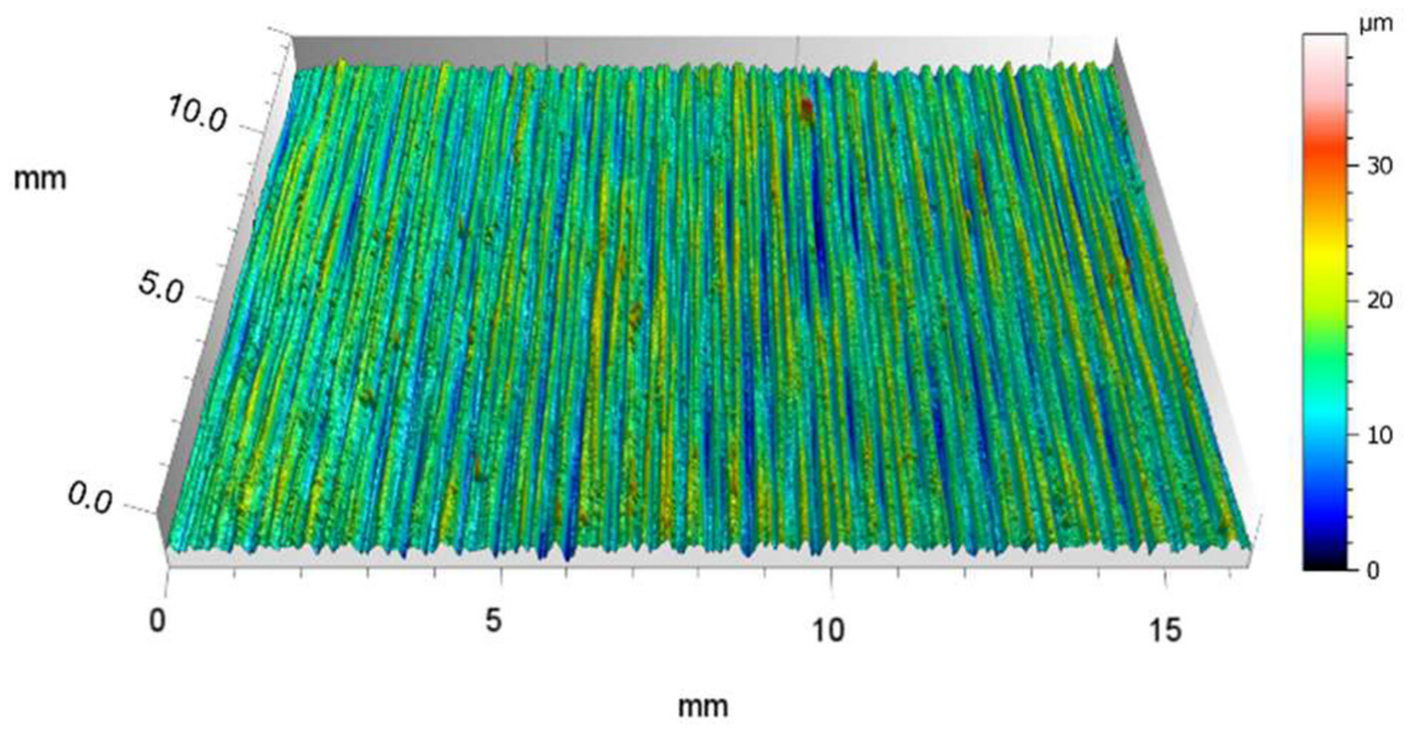
3D topography of the stone grind structure of the test skis. The surface roughness has an S_a_ value of 3.0 um, and the z-axis is amplified by 7%.

The silicone oils were purchased from Sigma Aldrich. The viscosity classification in centistokes refers to their viscosity at room temperature. The used oils had a viscosity of 10, 20, 50, and 100 cSt, respectively. Six test skis were supplied by Norwegian ski manufacturer Madshus and were identical 192 cm skate test skis with a medium grind with an S_a_ of 3.0 μm, as shown in Figure 1. Commercial fluorine-free spray-application glide waxes (HS6 and HS10) were supplied by Swix.

### Characterization and Measurements

The friction measurements were conducted in our full-scale linear ski tribometer. The construction and assessment of precision and accuracy of the tribometer were recently published by our group (Auganæs et al., 2022). The data acquisition system has been updated and now consists of a Compact RIO 9054 (National Instruments, USA) with a four-channel NI-9237 simultaneous bridge module connected to the amplified vertical and horizontal loads cells. An 8-meter magnetic encoder system (LM15, RLS Merilna tehnika, Slovenia) with a 50 μm resolution was connected to the NI-9411 digital module, and the data is transferred wirelessly to the computer in the control room. For this study, the adjustable sampling rate (0–10 kHz) was set to 5 kHz. For the friction testing, the ski speed was 6 m/s, with a 1.5 m sinusoidal-ramped acceleration profile to reduce vibrations. At each temperature, all skis were tested in three sets of three runs, i.e., all skis were first run 3 runs, then the entire series was repeated twice until *n* = 9 for every ski. This was done to distribute effects of track polishing across all the skis of the series.

The snow used for the linear tribometer was laboratory-grown dendritic snow, produced at −20 °C by evaporation-crystallization. The fresh snow was evenly distributed and compacted in the friction track and left to sinter for 16 h before starting the friction measurements. For the test on wet snow, aged snow was used. This snow was 1 week old laboratory-grown new snow which was sifted and distributed evenly in the tribometer by a steel blade and left to sinter for 16 h. On the day of testing, the cold room temperature was set to 5 °C 4 h before the testing started. The compaction of the snow surface before testing was done at 1 m/s by repeated runs at increasing normal load (50, 100, 200, 300, and 400 N). Then the track was worn in by increasing the speed by 1 m/s increments stepwise up to 6 m/s and lastly performing six repeated runs at 400 N at 6 m/s.

Snow humidity was measured with a Snow Moisture Meter Type 011 (DOSER Messtechnik GmbH & Co.KG, Germany) while the snow density was measured with the SLF Snow Sensor (FPGA Company GmbH, Switzerland). These measurements were conducted immediately after each experiment series, and the reported values are averages of five measurements equally spaced throughout the 3.5 meter measurement region. Contact angle measurements were recorded along the ski running direction with the MSA Flexible Liquid (KRÜSS GmbH, Germany) using distilled water.

To get a meaningful estimate of the glide performance of silicone oils, they were tested alongside a ski waxed with commercial liquid gliders suitable for each temperature. The term liquid glider refers to the state in which it is applied to the ski by aerosol spray. After application, the solvents evaporate, leaving a hard wax coating. One ski was left completely untreated in all measurements to establish the baseline glide performance of the sole material. This ski is referred to as “bare UHMWPE sole” or “reference.”

The application of the silicone oils was monitored by first weighing the amount of oil applied to the ski, and subsequently weighing every Fiberlene paper used to distribute and wipe the skis. By volume, 1 g of silicone oil which was distributed over the entire ski sole and left for 15 min. Then the sole was wiped with Fiberlene (Swix, Norway) paper until no more oil was visually removed, leaving an average of 50 mg of silicone oil, regardless of viscosity. Over the area of one ski, this equals 60 μg/cm^2^ or a 600 nm thick silicone oil layer assuming a uniform distribution. Before each friction test, the skis were placed in the cold lab for 1 h to equilibrate thermally to the testing temperature. Silicone oil was reapplied for each temperature. The liquid glide waxes were applied by spraying the ski, waiting 15 min for the solvent to evaporate then wiping off the sole with Fiberlene until no more wax was visually removed. While this is not the recommended application procedure for these products, it avoids mechanical brushing and ensures the structure of the ski remains comparable to the other skis.

## Results

The effect of silicone oil lubrication on ski-snow friction has been studied under three realistic snow conditions. Four silicone oils of different viscosity were tested alongside commercial products and benchmarked against an identical, untreated reference ski. Contact angle measurements and linear tribometer friction tests were used to assess the viability of liquid lubrication of skis on snow.

### Meteorological Parameters

The temperature and relative humidity of the air alongside the snow temperature, density, and humidity for the testing conditions are given in Table 1.

**Table 1:** Sets of meteorological and snow parameters measured for the various testing conditions.

**Air temp. [**°**C]**	**−10.0**	**−8.0**	**−1.0**	**5.0**
Snow temp [°C]	−10.6	−7.8	−1.0	0.0
Relative humidity [%]	50	-	62	73
Snow density [kg/m^3^]	317	407	340	814
Doser snow humidity [%]	13	16	17	73

### Contact Angle Measurements

**Figure 2:**
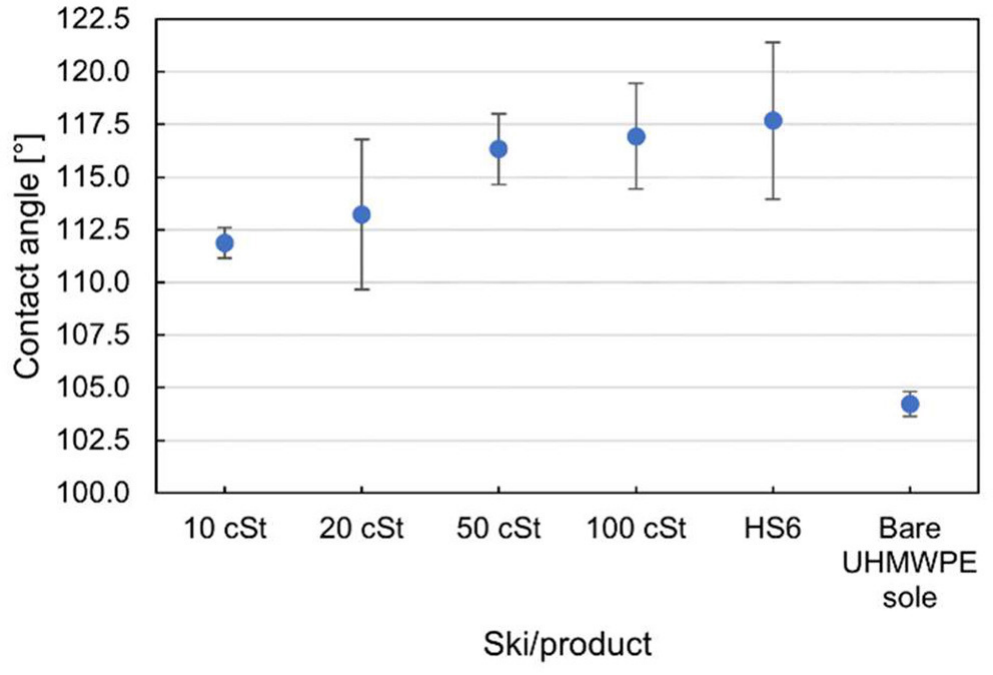
Contact angle measurements of the skis oiled with silicone oils, waxed with a commercial HS6 glide wax and the unwaxed reference ski. The contact angle is measured along the running direction, parallel to the stone grind of the base, and is averaged over ten measurements in the front and ten measurements in the rear contact zone of the skis. The error bars denote one standard deviation in each direction.

**Figure 3:**
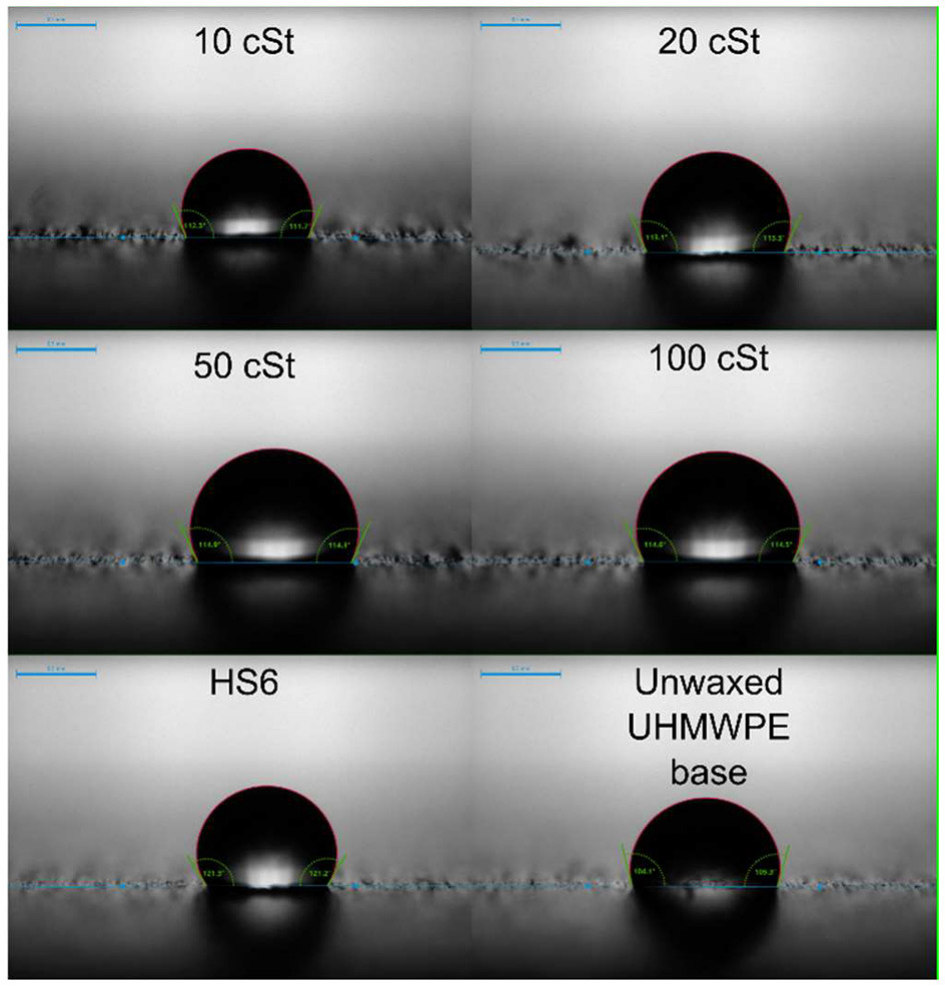
Selected images from the static water contact angle measurements.

The contact angle of water on a surface is a measure of hydrophobicity and is affected by the chemical properties of the material and the surface structure. In Figure 2, the contact angles of the oiled skis are plotted alongside the contact angle of a ski with a commercial wax and the unwaxed reference ski. Selected images from each series of measurements are presented in Figure 3.

### Friction Measurements

**Figure 4:**
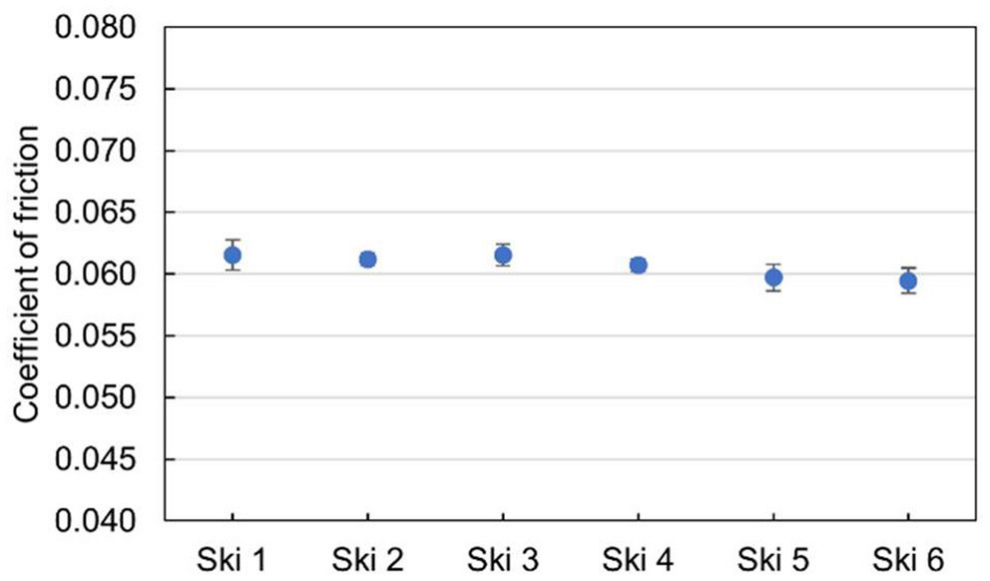
Baseline tests of the coefficient of friction for the six untreated test skis as received by the manufacturer, before treatment with oil or the reference wax.

To establish the six test skis used in the study were identical, a friction test was performed before treatment with oil or the reference wax. The temperature for this test was −8 °C, and the snow was fresh dendritic snow. The average coefficient of friction for the six skis was 0.0607 with a standard deviation of 0.009. The individual performances of each ski with corresponding error bars are given in Figure 4.

**Figure 5:**
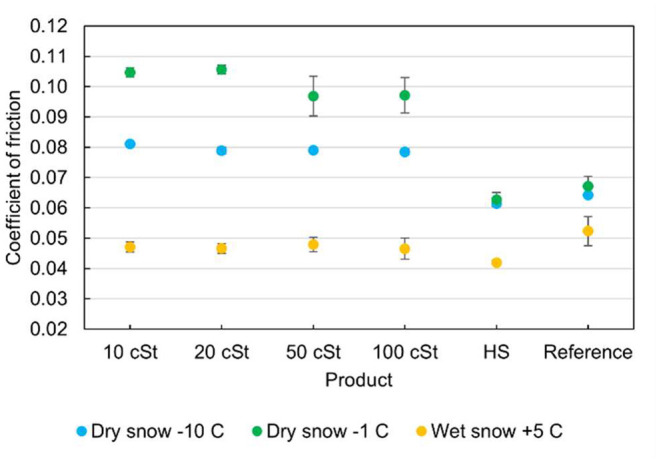
Coefficient of friction for measurements at −10, −1, and +5 °C.

The effect of the silicone oil treatments was tested at three temperatures and snow conditions ranging from −10 °C and dry snow to +5 °C wet snow. The average coefficients of friction at all temperatures are plotted in Figure 5 while the values and standard deviations are given in Table 2. At the lowest temperature of −10 °C, the silicone oils increased the coefficient of friction on average by 24% compared to the unwaxed reference ski. The liquid glide wax HS6 reduced the coefficient of friction by 5%, while the friction coefficient of the untreated reference ski was only marginally increased from the baseline test conducted at −8 °C. Among the silicone oils, there was a weak trend of reduced friction with increased viscosity.

**Table 2:** Coefficient of friction for the tribometer testing at −10, −1, and +5 °C.

**Ski**	**Treatment**	**Before treatment**	**After treatment**		
		**−8 **°**C**	**−10 **°**C**	**−1 **°**C**	**+5 **°**C**
1	10 cSt	0.062 ± 0.0012	0.081 ± 0.0009	0.105 ± 0.0015	0.047 ± 0.0016
2	20 cSt	0.061 ± 0.0005	0.079 ± 0.0011	0.106 ± 0.0014	0.047 ± 0.0016
3	50 cSt	0.062 ± 0.0009	0.079 ± 0.0008	0.097 ± 0.0066	0.048 ± 0.0023
4	100 cSt	0.061 ± 0.0005	0.079 ± 0.0011	0.097 ± 0.0059	0.046 ± 0.0035
5	HS6/10	0.060 ± 0.0011	0.061 ± 0.0011	0.063 ± 0.0024	0.042 ± 0.0006
6	None	0.059 ± 0.0010	0.064 ± 0.0006	0.067 ± 0.0031	0.052 ± 0.0048

*One standard deviation is reported. Oil or wax was reapplied for each temperature. Swix HS6 was used for −10 °C and HS10 for −1 and +5 °C*.

At the intermediate temperature of −1 °C the snow had more pre-existing water than at −10 °C, but still considered dry with a Doser snow humidity value of 17%. Under these conditions, the skis treated with silicone oils showed no benefit from a more hydrophobic surface and surprisingly increased the coefficient of friction by 44–57% while the commercial HS10 wax reduced the friction by 6%. Also at this temperature, the higher viscosity silicone oils were slightly better, albeit with higher standard deviations.

Under melting conditions (air temperature of 5 °C), all skis performed better than on colder dry snow, and here the silicone oils reduced the friction by about 10% compared to the untreated reference ski. The conventional liquid glider Swix HS10 reduced the friction by 20%.

## Discussion

### Contact Angle Measurements

The contact angles increased for all treatments compared to the bare UHMWPE reference, and the skis treated with the conventional glide wax HS6 had the highest contact angles. For the silicone oils, increased oil viscosity gives higher contact angles. This can be rationalized by the fact that higher viscosity silicone oils have higher surface tension, and therefore yield higher contact angles when in contact with water (Barca et al., 2014). Higher water contact angles means increased water repellence, which especially under wet conditions is favorable for reducing ski-snow friction.

### Friction Measurements

To measure the ski-snow friction and the effects of the different silicone oils, the skis were tested in a linear full-size ski tribometer (Auganæs et al., 2022). When the friction of the skis was tested prior to application of any products, we found the baseline performance to be practically identical. The six skis were therefore considered to be a good test set to investigate the effect of silicone oils. The average friction coefficient of the untreated skis of 0.060 represents, in our experience, skis with below average gliding properties and would therefore have potential for improvements.

At −10 °C, the silicone oils significantly increased the friction [*t*_(16)_ *p* < 0.001] while the conventional wax reduced the friction [*t*_(16)_ *p* = 0.001]. At these temperatures, there is little liquid water generated by frictional heating, causing a comparatively high portion of the friction to stem from solid-solid contact. Although the main function of a liquid lubricant is to reduce solid-solid contact, it also can induce a new source of resistance, namely shear forces in the lubrication film. In addition, the oil can contribute to an increase in the real contact area. The dry friction from solid ice-UHMWPE interactions is still relatively low when compared to systems such as stainless steel-UHMWPE (Yousif et al., 2010). Therefore it appears that the benefit of reducing solid-solid contact between the snow and UHMWPE by silicone oil lubrication gets overruled by the introduction of shear forces and increased contact area.

**Figure 6:**
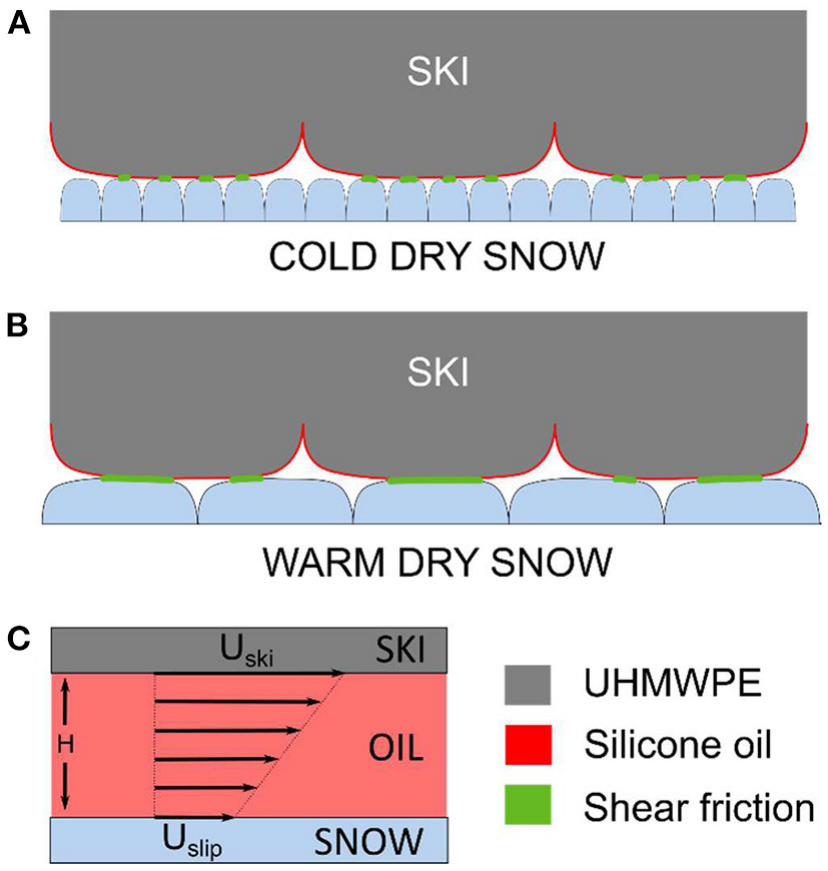
Shear friction contribution from liquid silicone oil lubricants illustrated for the two scenarios of **(A)** cold dry snow and **(B)** warm dry snow. The sum of shear friction is larger in the case of warm dry snow due to a larger real contact area. **(C)** Laminar flow velocity profile of the lubricated contact points used to estimate the shear friction.

A theoretical calculation of the magnitude of the shear forces is possible by assuming laminar flow in the silicone oil layer, see Figure 6C. The friction force from the shear stress within the lubricant layer, F_shear_, can be calculated with Equation 1, where τ is the shear stress, A the real contact area in m^2^, μ is the dynamic viscosity of the oil in N·s/m^2^ and H is the thickness of the silicone oil film in meters. U_ski_ is the ski speed relative to the snow in m/s and U_slip_ is the wall slip. So that the wall slip can be expressed as a fraction of the ski speed, we define U_slip_ = a · U_ski_, where a is the wall slip factor which can be set between 0 and 1.


$${\text{F}}_{\text{shear}}\text{= }\tau \cdot \text{A= }\frac{\mu \cdot ({\text{U}}_{\text{ski}}-\;{\text{U}}_{\text{slip}})\cdot \text{A}}{\text{H}}$$

To get an estimate for the shear friction some assumptions are required. The two contact zones of the loaded ski have a combined length of 43 cm and the ski width is 4.4 cm, dictating the apparent contact area to be 189 cm^2^. The real contact area (of ski in contact with snow during sliding) is assumed to be 1% of the apparent contact area (Theile et al., 2009; Mössner et al., 2021). While the low real contact area is a result of the coarseness of the snow and the structured ski surface, both surfaces are assumed perfectly flat in the points of contact. The dynamic viscosity of a 100 cSt silicone oil with a density of ~1 g/cm^3^ converts to 0.1 N·s/m^2^, and the thickness of the oil layer is calculated from the amount of oil applied on the ski and is 600 nm. Under noslip conditions, which is highly unlikely considering the icephobic nature of silicone oils, the calculated shear force is 190 N. This corresponds to a very high friction coefficient of 0.475, meaning this system must have significant wall-slip. Starting with a realistic friction coefficient for this system of 0.08, the shear force would equate to 32 N assuming shear is the sole resistance mechanism, and this would require a wall-slip factor of 0.83.

These calculations show that viscous shear in oil films of realistic thicknesses can quickly lead to high resistance forces between skis and snow. As no change in friction coefficient was observed over the course of the experiments, it is reasonable to conclude the oil did not wear away at a significant rate, and consequently the wall-slip factor must be high. Furthermore, as the shear force calculation is proportional to viscosity it is intriguing that the measured total friction appears not to be. This can be explained by the higher viscosity oils having a higher degree of wall-slip causing the two contributions cancel each other.

Bearing in mind the friction values of the untreated and commercial wax skis were practically unchanged compared to the values at −10 °C, the considerable increase for the silicone oil skis at −1 °C is intriguing, and contrary to common conceptions in ski waxing.

Our explanation of this notable friction increase on warm dry snow stems from changes in the snow. Cold dry snow has sharper edges and slower metamorphism compared to warmer snow, which means the snow in the experiment at −1 °C has more rounded features. In turn, this means the contact area of a ski sliding over warm snow can be larger than on cold snow. The shear forces from the liquid silicone lubricants are proportional to the real contact area between the ski and the snow. The two scenarios are illustrated in Figures 6A,B. The commercial HS waxes are considerably harder coatings than pure silicone oils, and therefore no detrimental effects due to shear friction were observed.

The competitive advantage of fluorinated glide products is largest when the snow humidity is high, and the silicone oils did significantly reduce the ski-snow friction under these conditions [*t*_(16)_ *p* < 0.025] compared to the untreated ski. This can be attributed to the increased hydrophobicity of the sole material, greatly reducing the friction contribution from capillary suction. The static water contact angle of the conventional wax was even higher than for the silicone oils. In combination with the lack of viscous shear, the conventional HS waxes outperformed the silicone oils also on the warmest and wettest of skiing conditions.

**Figure 7:**
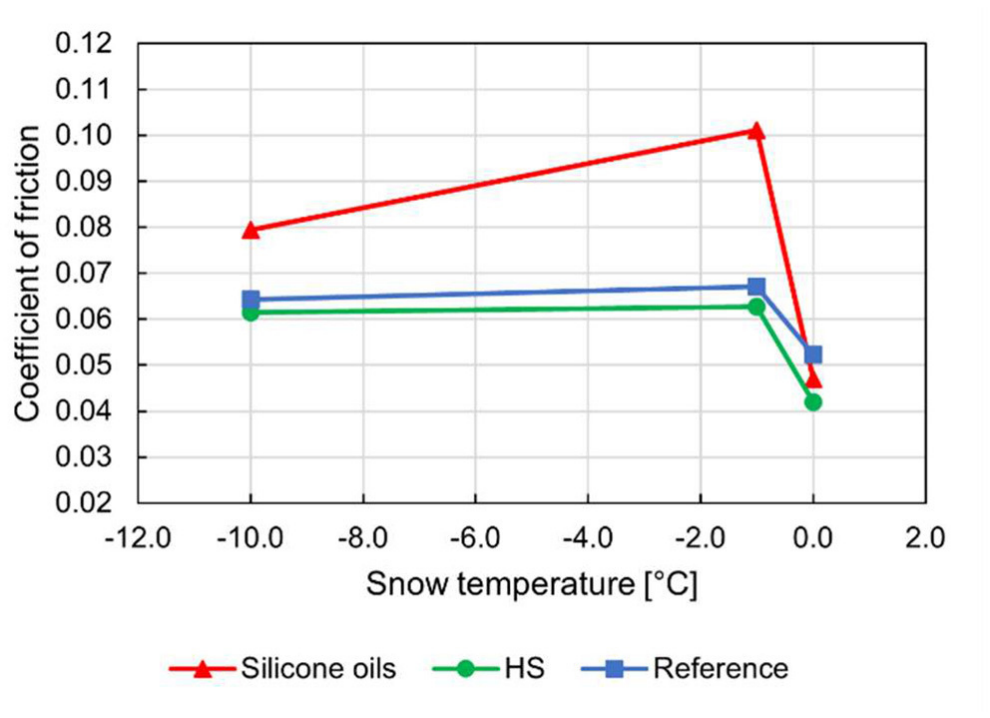
Coefficient of friction vs. snow temperature for the skis treated with silicone oils, the HS waxed skis and the bare UHMWPE sole reference skis.

While the air temperature was 5 °C during these tests, the snow, which is undergoing a phase change, maintained 0 °C throughout the experiment. While all cold snow is relatively dry, snow around 0 °C can vary greatly in humidity. This is often also the case during competitions, where racers pass through colder shaded parts of the track as well as sun-soaked areas. Consequently, glide products need to be highly specialized for specific conditions to give a competitive advantage while simultaneously maintaining a good baseline performance when outside the ideal temperature or humidity window. For the case of pure silicone oils, this performance window appears to contain only warm temperatures and humid snow, as shown in Figure 7. In the authors' opinion, it is unlikely that liquid silicone oil lubricants will have a significant potential for UHMWPE-snow lubrication.

On the other hand, by employing silicone oils as additives in other coating matrices one may be able to retain the wet snow properties from the silicone oils while avoiding the detrimental effects under dry conditions. As the effects in this study were obtained with very low quantities of oil, we consider the viability of silicone oils as additives as realistic.

## Conclusion

Silicones possess a lot of the properties which are desirable for a viable replacement for fluorinated glide waxes, such as high hydrophobicity and low toxicity. In this study of liquid oil lubrication of cross-country skis, we have tested the simplest of silicone oils in a range of viscosities and found the effect on friction to vary widely with the snow conditions and temperature. On dry snow, the liquid lubricants were found to significantly increase friction compared to commercial gliders and the untreated reference ski. The effect was larger on warmer and more transformed snow than on cold snow. This led to the conclusion that the use of liquid silicone oil lubricants introduces a shear force contribution to the friction which is dependent on the real contact area and therefore more pronounced on warmer dry snow with rounder features. Under wet conditions, the hydrophobic contribution from the silicone oils reduced the friction by 10% compared to the untreated reference ski, supported by contact angle measurements.

The findings suggest that liquid lubrication of ski-snow interactions by these silicone oils has a very narrow performance window. However, by virtue of their properties on wet snow, these or other silicone compounds may serve as valuable components or additives in the next generation of fluorine-free glide products for skis. Further work on optimization of oil properties is likely to widen the scope, and according to the presented theory on shear-induced friction, silicones of both higher and lower viscosity may hold performance benefits to the ski-snow tribological system. New non-toxic glide products with performance rivaling that of fluorinated products will not only save valuable seconds in competitions, but also improve the health of waxers and reduce the ecological impact from ski wax.

## Data Availability Statement

The raw data supporting the conclusions of this article will be made available by the authors, without undue reservation.

## Author Contributions

AB designed the study, conducted the experimental work, and wrote the manuscript. SA and AK-P contributed to scientific discussions and reviewed the manuscript. All authors contributed to the article and approved the submitted version.

## Funding

AB and SA are funded through the Nano2Glide IPN project granted by the Norwegian Research Council, Project No. 296540.

## Conflict of Interest

The authors declare that the research was conducted in the absence of any commercial or financial relationships that could be construed as a potential conflict of interest.

## Publisher's Note

All claims expressed in this article are solely those of the authors and do not necessarily represent those of their affiliated organizations, or those of the publisher, the editors and the reviewers. Any product that may be evaluated in this article, or claim that may be made by its manufacturer, is not guaranteed or endorsed by the publisher.
