# Corrigendum: Effect of polydimethylsiloxane oil lubrication on the friction of cross-country UHMWPE ski bases on snow

**DOI:** 10.3389/fspor.2023.1203992

**Published:** 2023-04-28

**Authors:** Audun Formo Buene, Sondre Bergtun Auganæs, Alex Klein-Paste

**Affiliations:** Department of Civil and Environmental Engineering, Norwegian University of Science and Technology, Trondheim, Norway

**Keywords:** silicone oils, ski-snow friction, linear tribometer, skiing, lubrication

A corrigendum on Effect of polydimethylsiloxane oil lubrication on the friction of cross-country UHMWPE ski bases on snow Buene AF, Auganæs SB and Klein-Paste A. (2022) Front. Sports Act. Living 4:894250. doi: 10.3389/fspor.2022.894250

**Text Correction**

In the published article, there was an error. The average amount of applied silicone oil to each ski was reported to be 5 mg, while the correct amount was 50 mg.

A correction has been made to **Materials and Methods**, *Characterization and Measurements,* paragraph 5. The sentence previously stated:

“[…] Then the sole was wiped with Fiberlene (Swix, Norway) paper until no more oil was visually removed, leaving an average of 5 mg of silicone oil, regardless of viscosity. Over the area of one ski, this equals 6 µg/cm^2^ or a 60 nm thick silicone oil layer assuming a uniform distribution. […]”

The corrected sentence appears below:

“[…] Then the sole was wiped with Fiberlene (Swix, Norway) paper until no more oil was visually removed, leaving an average of 50 mg of silicone oil, regardless of viscosity. Over the area of one ski, this equals 60 µg/cm^2^ or a 600 nm thick silicone oil layer assuming a uniform distribution. […]”

A correction has been made to **Discussion**, *Friction measurements,* paragraph 4. The sentence previously stated:

“[…] the thickness of the oil layer is calculated from the amount of oil applied on the ski and is 60 nm. Under no-slip conditions, which is highly unlikely considering the icephobic nature of silicone oils, the calculated shear force is 1.90 kN. This corresponds to a very high friction coefficient of 4.75, meaning this system must have significant wall-slip. Starting with a realistic friction coefficient for this system of 0.08, the shear force would equate to 32 N assuming shear is the sole resistance mechanism, and this would require a wall-slip factor of 0.98.”

The corrected sentence appears below:

“[…] the thickness of the oil layer is calculated from the amount of oil applied on the ski and is 600 nm. Under no-slip conditions, which is highly unlikely considering the icephobic nature of silicone oils, the calculated shear force is 190 N. This corresponds to a very high friction coefficient of 0.475, meaning this system must have significant wall-slip. Starting with a realistic friction coefficient for this system of 0.08, the shear force would equate to 32 N assuming shear is the sole resistance mechanism, and this would require a wall-slip factor of 0.83.”

The authors apologize for this error and state that this does not change the scientific conclusions of the article in any way. The original article has been updated.

